# Molecular Mechanisms, Dynamic Lesions, and Therapeutic Targets in Intestinal Ischemia–Reperfusion Injury: A Systematic Review

**DOI:** 10.3390/ijms27041763

**Published:** 2026-02-12

**Authors:** Julia Marton, Răzvan Alexandru Ciocan, Ioana Bâldea, Mădălina Luciana Gherman, Dan Gheban, Adriana Filip, Ionuț Răzvan Pașcalău, Florin Vasile Mihăileanu, Raluca Maria Pop, Claudia Diana Gherman

**Affiliations:** 1 Department of Anatomy and Embryology, “Iuliu Hațieganu” University of Medicine and Pharmacy, 400012 Cluj-Napoca, Romania; marton.julia@elearn.umfcluj.ro (J.M.); gabriela.filip@umfcluj.ro (A.F.); 2 Department of Surgery—Practical Abilities, “Iuliu Hațieganu” University of Medicine and Pharmacy, 400337 Cluj-Napoca, Romania; 3 Department of Physiology, “Iuliu Haţieganu” University of Medicine and Pharmacy, 400006 Cluj-Napoca, Romania; 4 Experimental Centre, “Iuliu Haţieganu” University of Medicine and Pharmacy, 400349 Cluj-Napoca, Romania; 5 Department of Pathology, “Iuliu Hațieganu” University of Medicine and Pharmacy, 400012 Cluj-Napoca, Romania; 6 Department of Surgery, Surgery II “Iuliu Hațieganu” University of Medicine and Pharmacy, 400006 Cluj-Napoca, Romania; 7 Department of Pharmacology, Toxicology and Clinical Pharmacology—Morphofunctional Sciences, “Iuliu Haţieganu” University of Medicine and Pharmacy, 400012 Cluj-Napoca, Romania; raluca.pop@umfcluj.ro

**Keywords:** intestinal ischemia–reperfusion, oxidative stress, NF-κB, Nrf2, endothelial dysfunction, gut microbiota, dexmedetomidine, ischemic conditioning, hyperbaric oxygen, tight junctions, molecular pathways

## Abstract

Intestinal ischemia–reperfusion injury (IRI) represents a major cause of morbidity and mortality in abdominal surgery, trauma, and intestinal transplantation. The pathophysiological process involves a biphasic cascade that begins with ischemic hypoxia and progresses to amplified cellular and molecular injury upon reperfusion. This review synthesizes recent mechanistic insights regarding endothelial and microvascular dysfunction, epithelial barrier breakdown, microbiota-driven systemic propagation, and the involvement of oxidative/nitrosative stress and inflammatory signaling. The novelty of our review’s approach is the focus on experimental and translational studies and correlation of the data with future directions for mechanistic research and clinical implementation. Despite promising preclinical results, heterogeneity in study protocols or/and model limitations make clinical translation challenging. Recent studies have demonstrated that mitochondria, tight junction proteins, adhesion molecules and innate immune receptors are critical determinants of lesion evolution. Based on these, the current therapeutic strategies include antioxidants, adenosine pathway modulators, dexmedetomidine, ischemic conditioning, hyperbaric oxygen therapy, and microbiota-targeted interventions. Since each mechanism is acting on distinct molecular pathways, a multimodal therapy that integrates redox modulation, endothelial protection, microbiome regulation, and the identification and employment of precision biomarkers is likely to improve outcomes. Beyond summarizing established molecular mechanisms, this review critically reassesses why decades of promising experimental strategies for intestinal ischemia–reperfusion injury has largely failed to translate into effective clinical therapies. By distinguishing context-dependent mechanisms from pathways with consistent translational relevance, we highlight key methodological and biological barriers limiting clinical applicability. Furthermore, we propose a temporally structured, multimodal therapeutic framework that integrates phase-specific pathophysiology with targeted interventions, aiming to inform future experimental design and improve translational success.

## 1. Introduction

Intestinal ischemia–reperfusion injury (IRI) is a significant clinical condition observed during abdominal surgery, intestinal transplantation, major vascular reconstruction, strangulated hernias, volvulus, and acute mesenteric ischemia. The initial events are represented by an abrupt disruption and subsequent restoration of mesenteric blood flow, which initiates a series of molecular and structural alterations [1,2,3]. IRI has a biphasic pattern consisting of (1) an ischemia phase, when hypoxia and nutrient deficiency lead to metabolic failure, followed by (2) reperfusion, a paradoxical process in which reoxygenation worsens tissue damage instead of restoring physiological equilibrium [1].

**Figure 1:**
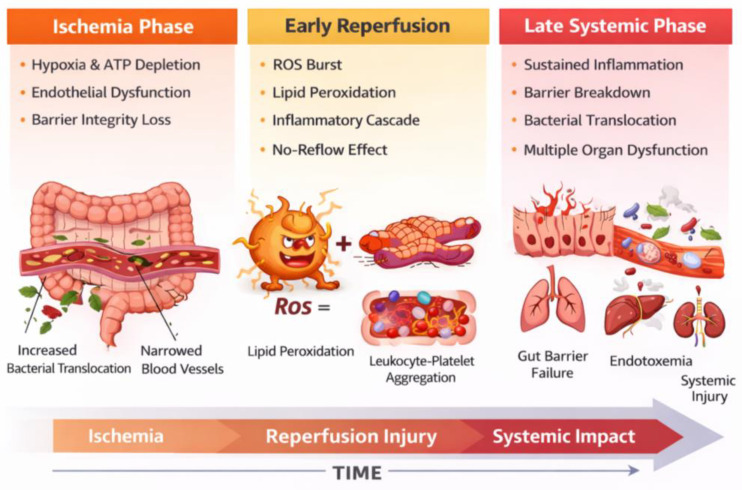
Temporal model of intestinal ischemia–reperfusion injury. (Schematic representation of the dynamic progression from ischemia to early reperfusion and late systemic inflammation, illustrating phase-specific mechanisms including metabolic failure, oxidative stress, microvascular dysfunction, epithelial barrier disruption, and immune activation. The model emphasizes the temporal heterogeneity of injury pathways and the need for phase-adapted therapeutic strategies, executed with the aid of Adobe Photoshop version 26, released October 2024 (accessed online on 1 December)).

Ischemia leads to rapid ATP depletion, which disrupts ionic transporters, destabilizes cytoskeletal framework, and causes epithelial cell swelling, apoptosis, and necrosis. Mitochondrial dysfunction is a critical element, since inhibition of oxidative phosphorylation hastens metabolic deterioration [2,4]. Reperfusion triggers an increase in reactive oxygen species (ROS) and reactive nitrogen species (RNS), including nitric oxide and arachidonic acid metabolites. These lead to oxidative stress-induced damage, complement activation, and the secretion of powerful pro-inflammatory cytokines such as TNF-α (tumor necrosis factor-α), IL-1β (interleukin 1-β), and IL-6 (interleukin 6) [5,6,7] (Figure 1). These cytokines exacerbate both local and systemic inflammation, which can eventually progress to systemic inflammatory response syndrome (SIRS) and multiple organ dysfunction syndrome (MODS).

Figure 1 presents a temporal injury model of intestinal ischemia–reperfusion, delineating the ischemic phase, early reperfusion, and late systemic phase, and highlighting the dominant molecular mechanisms and injury drivers at each stage.

A fundamental IRI attribute is the compromise of the intestinal epithelial barrier. Structural proteins that maintain tight junctions, such as occludin, claudin-1, and zonula occludens-1 (ZO-1), undergo early degradation after reperfusion, leading to heightened mucosal permeability, and facilitate bacterial translocation and endotoxemia [3,4,8,9]. At the same time, the microvascular dysfunction characterized by endothelial edema, leukocyte adhesion, platelet aggregation, and impaired capillary perfusion contributes to persistent tissue hypoxia despite revascularization attempts [5,10].

Recent research highlights the essential role of the gut microbiota–immune axis in the transition from localized damage to systemic involvement. Ischemia and reperfusion rapidly induce dysbiosis, alter mucin composition, hinder epithelial regeneration, and activate Toll-like receptor (TLR) pathways together with NLRP3 inflammasome signaling. As a result, cytokine production is enhanced and tissue damage is strongly exacerbated [1,6,7,8,9,11].

Despite increasing comprehension of the molecular mechanisms involved in IRI, such as the activation of NF-κB (nuclear factor k B) and AP-1 (activator protein 1) pathways, endothelial dysfunction driven by endothelin-1 and adhesion molecules, excessive mitochondrial production of ROS, apoptosis induction, and dysregulation of autophagy, the application of these insights to develop effective therapeutic interventions remains a considerable challenge [12,13,14].

This review aims to summarize the recent mechanistic insights into endothelial and microvascular dysfunction, epithelial barrier disruption, microbiota-driven systemic effects, and oxidative/nitrosative stress-mediated inflammation. The novelty of our approach lies in emphasizing experimental and translational studies while linking current evidence to future mechanistic research and clinical applications. Although preclinical findings are promising, variability in models and protocols limits clinical translation. Recent work has identified mitochondria, tight junction proteins, adhesion molecules, and innate immune receptors as key drivers of lesion progression. Accordingly, current pharmacological therapies, including antioxidants (e.g., N-acetylcysteine and melatonin), free radical scavengers, vasodilators, anti-inflammatory drugs, and adenosine receptor modulators, have shown significant protective benefits in animal models [10,11,12]. Non-pharmacological interventions, including ischemia preconditioning, hyperbaric oxygen treatment (HBOT), and controlled hypothermia, have considerable benefits in alleviating oxidative stress, neutrophil infiltration, and microvascular dysfunction [10,11,12]. Moreover, therapies aimed at microbiota, including probiotics and short-chain fatty acid (SCFA) supplementation, have surfaced as potential strategies for restoring epithelial integrity and moderating systemic inflammation [12,13,14,15,16]. As a first original element of the current review, we propose a comprehensive evaluation of the molecular, biochemical, structural, and microbiological processes involved in intestinal ischemia–reperfusion injury as a start to developing multimodal, mechanistic-based therapeutic strategies. Secondly, the review evaluates the efficacy of using therapies targeting distinct IRI-involved pathways on different preclinical models with translational applicability in abdominal and transplant surgery. Specifically, the present work aims to critically reassess intestinal ischemia–reperfusion injury reports and to evaluate why promising experimental interventions have repeatedly failed to demonstrate clinical benefit. We will address the differences between in vitro, in vivo, ex vivo, and human data contributions to overinterpretation. Moreover, we will evaluate which mechanistic pathways may be context-dependent rather than universally relevant. By reframing intestinal IRI as a temporally dynamic and multifactorial process, this review seeks to move beyond descriptive synthesis toward a conceptually reorganized, clinically actionable framework.

Unlike previous reviews that primarily provide comprehensive catalogs of molecular pathways and therapeutic targets, the present work aims to critically re-evaluate intestinal ischemia–reperfusion injury from a translational perspective. Specifically, we examine why interventions that demonstrate robust efficacy in experimental models frequently fail to confer benefit in clinical settings, and which mechanistic pathways may be context-dependent rather than universally actionable. By integrating experimental, ex vivo, and limited clinical evidence, this review seeks to identify translational bottlenecks, clarify unresolved controversies, and reorganize existing knowledge into a temporally structured, multimodal therapeutic framework. This approach is intended not only to summarize current understanding, but to guide future research toward strategies with greater clinical relevance and impact.

To enhance conceptual clarity and move beyond descriptive synthesis, this review incorporates two original conceptual figures that reorganize existing mechanistic knowledge into clinically relevant frameworks. Rather than introducing new experimental data, these figures integrate established evidence to illustrate temporal injury evolution and therapeutic decision-making in intestinal ischemia–reperfusion injury. This approach is intended to facilitate interpretation, highlight translational bottlenecks, and support a phase-specific, multimodal treatment paradigm.

## 2. Materials and Methods

### 2.1. Study Design

This review was conducted as a structured, narrative, integrative synthesis of experimental, translational, and clinical evidence concerning intestinal ischemia–reperfusion injury. The methodological approach followed PRISMA 2020 recommendations for transparent reporting of review processes, while maintaining a narrative format appropriate for mechanistic and molecular analyses where formal meta-analysis is not feasible due to heterogeneity in study models [17].

The objective was to integrate histological, biochemical, molecular, and microbiological findings with therapeutic strategies described across the current literature, thereby identifying convergent mechanisms and translational opportunities.

### 2.2. Literature Search Strategy

A comprehensive literature search was performed across five major scientific databases—PubMed, Scopus, Web of Science, Google Scholar, and Embase—from 1 January 2000 to 31 December 2024 (Internet Archive link: https://archive.org/details/osf-registrations-5qpyu-v1, Registration DOI: 10.17605/OSF.IO/5QPYU).

Search terms included combinations of:

- “Intestinal ischemia reperfusion”;
- “Intestinal ischemia reperfusion injury”;
- “Oxidative stress AND intestine”;
- “Intestinal barrier”;
- “Tight junctions ischemia”;
- “Microcirculation intestine I/R”;
- “Gut microbiota AND ischemia reperfusion”;
- “Ischemic conditioning intestine”;
- “Hyperbaric oxygen AND intestinal I/R”;
- “Dexmedetomidine AND I/R”.

Boolean operators (AND/OR), MeSH terms, and truncated forms were applied to maximize coverage.

Reference lists of included publications were further screened for additional relevant studies (snowball strategy).

### 2.3. Eligibility Criteria

Studies were included if they met all the following criteria:

1. Investigated intestinal ischemia–reperfusion injury, using:
  ○ In vivo animal models;
  ○ Ex vivo perfusion models;
  ○ Clinical or perioperative human data;
  ○ Molecular in vitro studies directly related to intestinal I/R mechanisms.
2. Reported at least one of the following outcomes:
  ○ Histological injury scores;
  ○ Oxidative stress markers (MDA, SOD, CAT, GSH-Px);
  ○ Inflammatory cytokines (TNF-α, IL-1β, IL-6);
  ○ Tight junction protein expression;
  ○ Microvascular flow parameters;
  ○ Mitochondrial structure/function;
  ○ Molecular pathway activation;
  ○ Therapeutic intervention outcomes.
3. Were published in peer-reviewed journals in English.

Exclusion criteria:

- Studies not directly related to intestinal tissue (e.g., isolated hepatic/cerebral I/R);
- Insufficient methodological description;
- Conference abstracts without full text;
- Reviews lacking primary data (excluded from synthesis but used for contextual interpretation).

### 2.4. Data Extraction

For each eligible study, the following data were extracted independently by two reviewers:

- Experimental organism and characteristics;
- Ischemia duration and type (occlusive, non-occlusive, SMA ligation);
- Reperfusion period;
- Tissue sampling techniques;
- Biomarkers of oxidative stress and inflammation;
- Molecular pathways assessed (e.g., NF-κB, PI3K/Akt, MAPK, Nrf2);
- Histopathological scoring systems;
- Measurement of tight junction proteins (occludin, claudin-1, ZO-1);
- Microcirculation assessments (laser Doppler, intravital microscopy);
- Therapeutic agents and dosing protocols;
- Probiotic strains and microbiota analysis methods;
- Primary outcomes and reported statistical significance.

**Figure 2:**
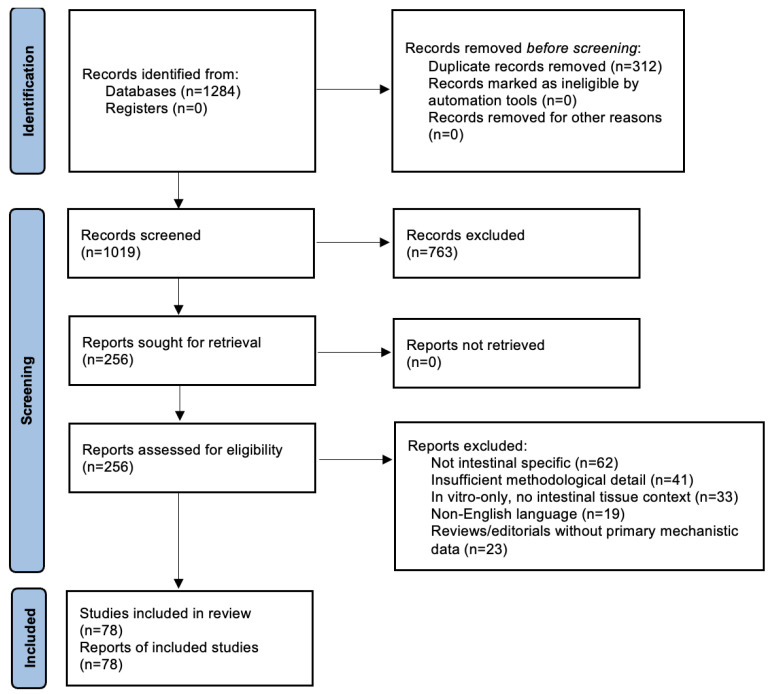
Flow diagram depicting the identification of studies via databases and registers.

Discrepancies were resolved through consensus (Figure 2).

### 2.5. Quality Assessment

Studies involving animal models were assessed using the SYRCLE risk-of-bias tool, allowing evaluation across domains such as randomization, blinding, allocation, attrition, and selective reporting [18]. Human observational studies were evaluated with the Newcastle–Ottawa Scale (NOS), and systematic reviews referenced during interpretation were appraised using AMSTAR-2 guidelines [19].

Only studies achieving moderate or high methodological quality were included in the core mechanistic synthesis.

### 2.6. Data Synthesis and Analytical Framework

Due to heterogeneity in model designs—differences in ischemia time (20–120 min), reperfusion duration (30 min–24 h), species, surgical technique, temperature control, and outcome markers—a meta-analysis was not appropriate.

Thus, results were synthesized according to five mechanistic domains, enabling structured interpretation:

1. Microcirculatory and endothelial dysfunction;
2. Oxidative and nitrosative stress pathways;
3. Inflammatory and immune signaling cascades;
4. Epithelial barrier structure and permeability;
5. Gut microbiota modulation and systemic propagation.

Therapeutic strategies were grouped into pharmacological, microbiota-targeted, and conditioning-based interventions.

This approach enabled direct comparison across studies with different experimental frameworks while maintaining mechanistic coherence.

## 3. Pathways Involved in IRI Development

Intestinal ischemia–reperfusion injury is a complex pathophysiological process including oxidative stress, inflammatory pathways, endothelial dysfunction, epithelial barrier disruption, and changes in gut microbiota. This section integrates histological, biochemical, and molecular data to elucidate the intersection of these processes in causing increasing mucosal and systemic damage.

### 3.1. Microvascular Injury, Epithelial Barrier Failure, and Lesion Evolution

Ischemia initiates a rapid metabolic collapse in intestinal tissues, primarily driven by ATP depletion and mitochondrial dysfunction. Across experimental and translational studies, intestinal ischemia–reperfusion injury is consistently characterized by profound microvascular dysfunction that precedes and drives epithelial barrier breakdown (Table 1).

Reperfusion does not immediately restore microcirculatory perfusion; instead, endothelial swelling, leukocyte adhesion, and platelet aggregation contribute to persistent capillary obstruction and the no-reflow phenomenon, sustaining tissue hypoxia despite revascularization [16,20]. The ‘no-reflow phenomenon’ refers to the failure of adequate microvascular perfusion despite restoration of macroscopic blood flow following reperfusion [21].

A notable finding in experimental models is that the extent of reperfusion injury sometimes exceeds the original ischemia damage. Reperfusion induces abrupt reoxygenation, resulting in the overproduction of mitochondrial reactive oxygen species, activation of xanthine oxidase, and heightened NADPH oxidase activity, thereby commencing a self-perpetuating oxidative cascade [22,23]. The natural antioxidant systems, including superoxide dismutase, catalase, glutathione peroxidase, and Nrf2 signaling responses are insufficient, which allows oxidative damage to advance [24,25].

The inflammatory response is intricately linked to oxidative damage. Reactive oxygen species (ROS) activate transcription factors including NF-κB and AP-1, leading to the production of TNF-α, IL-1β, and IL-6, which subsequently promote leukocyte recruitment, complement activation, and microvascular blockage. Increase in adhesion molecules (ICAM-1, VCAM-1, P-selectin) exacerbates the no-reflow phenomena, consequently undermining mucosal perfusion even when arterial flow is restored [26,27].

Cytoskeletal disorganization and degradation of tight junction proteins—including occludin, claudin-1, and ZO-1—facilitate paracellular permeability and epithelial detachment, amplify mucosal vulnerability [20,21,28] and increase IRI severity [29,30]. Histologically, reperfusion accelerates the development of subepithelial edema, hemorrhage, and dense neutrophil infiltration. Electron microscopy frequently reveals mitochondrial injury during IRI such as mitochondrial swelling, cristae fragmentation, and structural collapse [22,31].

Proteomics-based investigations demonstrate compensatory upregulation of stress-responsive pathways, including heat shock proteins HSP70 and HSP90, antioxidant enzymes, and redox regulators, which suggests active cellular attempts to restore homeostasis and limit injury propagation [23,32].

### 3.2. Oxidative Stress and Inflammatory Signaling

Reperfusion exacerbates intestinal injury through a burst of reactive oxygen species (ROS) driven predominantly by mitochondrial ROS overproduction, xanthine oxidase activation, and NADPH oxidase activity and activation of pro-inflammatory signaling pathways. These pathways activate transcription factors such as NF-κB and AP-1, leading to robust cytokine release (TNF-α, IL-1β, IL-6) and amplification of the inflammatory cascade (Table 2) [24,33].

Complement activation and increased expression of adhesion molecules ICAM-1 and VCAM-1 intensify leukocyte recruitment and microvascular plugging [25]. The endogenous antioxidant response, including superoxide dismutase (SOD), catalase (CAT), and glutathione peroxidase (GPX) is insufficient to neutralize the accumulated oxidative load, failing to prevent lipid peroxidation and cellular apoptosis [26,34,35].

Adenosine receptor-mediated signaling plays an important immunoregulatory role, modulating cytokine release, leukocyte adhesion, and mucosal perfusion. Modulation of A_2_A receptor pathways has been shown to attenuate oxidative stress and reduce inflammatory infiltration in multiple experimental models [27,36,37].

Although oxidative stress and NF-κB-dependent inflammatory signaling are consistently activated during intestinal ischemia–reperfusion injury, their ubiquitous involvement across experimental models suggests limited mechanistic specificity. Rather than acting as isolated drivers, redox imbalance and inflammatory activation function as upstream amplifiers that modulate downstream processes, including microcirculatory dysfunction, epithelial barrier failure, and regulated cell death. This perspective helps explain why therapeutic strategies targeting single inflammatory or oxidative mediators have shown limited clinical efficacy, underscoring the need for integrative approaches that address interacting injury domains rather than isolated pathways [36,37].

Given their consistent activation across virtually all experimental models of intestinal ischemia–reperfusion injury, oxidative stress and NF-κB signaling should be viewed less as discrete therapeutic targets and more as upstream amplifiers that modulate downstream processes. This lack of mechanistic specificity may partly explain the limited clinical efficacy of broad antioxidant or anti-inflammatory strategies, reinforcing the need to focus on context-dependent and phase-specific interventions.

### 3.3. Gut Microbiota–Immune Axis and Systemic Propagation of Injury

Disruption of the intestinal epithelial barrier represents a central event linking local ischemic injury to systemic inflammatory responses. Tight junction proteins, such as occludin, claudin-1, and ZO-1, are rapidly degraded after reperfusion, leading to increased mucosal permeability, bacterial translocation, and endotoxemia [38,39,40]. These mechanisms induce systemic inflammatory response syndrome (SIRS) and contribute to secondary damage in distant organs, such as the lungs and brain [39], leading to multiorgan dysfunction [31,38,41].

Loss of barrier function leads to bacterial translocation, luminal endotoxin leakage, and systemic immune activation. Recent evidence has demonstrated that microbiota composition and its metabolites, especially short-chain fatty acids (SCFAs), play critical roles in modulating epithelial resilience and systemic inflammatory outcomes [28,29,39,40]. Dysbiosis, reduced levels of beneficial short-chain fatty acids (SCFAs), and depletion of mucin during IRI enhance TLR4 and NLRP3 inflammasome activation, promote cytokine escalation, further compromise barrier integrity and impede healing mechanisms [40]. Probiotic supplementation, conversely, has been shown to reduce inflammatory cytokines, improve villus morphology, restore tight junction proteins, and enhance survival in preclinical models [30,41,42,43].

Importantly, the pathophysiological processes activated during intestinal ischemia–reperfusion injury do not occur in isolation. Microcirculatory impairment, epithelial barrier disruption, inflammatory amplification, and regulated cell death evolve as interdependent processes that reinforce one another across distinct temporal phases of injury. Recognizing these interactions is essential for understanding injury propagation and for identifying therapeutic strategies with translational relevance [41,44].

### 3.4. Apoptosis, Ferroptosis and Pyroptosis Mechanisms of Intestinal Cell Death

Regulated cell death represents a central convergence point linking oxidative stress, inflammation, microvascular dysfunction, and epithelial barrier failure in intestinal ischemia–reperfusion injury. Rather than functioning as independent pathways, apoptosis, ferroptosis, and pyroptosis interact dynamically, sharing upstream triggers and downstream consequences that collectively shape tissue injury and systemic inflammatory responses [44].

Intestinal ischemia–reperfusion injury is characterized by the activation of multiple, tightly regulated cell death pathways that collectively contribute to epithelial loss, barrier dysfunction, and systemic inflammation. Among these, apoptosis, ferroptosis, and pyroptosis represent the most extensively documented and mechanistically relevant forms of regulated cell death in intestinal epithelial and endothelial cells during IRI [44,45].

Collectively, apoptosis, ferroptosis, and pyroptosis form a tightly interconnected cell-death network in intestinal ischemia–reperfusion injury. Oxidative stress and mitochondrial dysfunction act as shared upstream drivers, while inflammatory mediators released during pyroptosis further sensitize surrounding cells to apoptotic and ferroptotic death. In turn, epithelial loss and barrier disruption amplify microbial translocation and immune activation, reinforcing injury propagation [45,46]. This crosstalk highlights the limitations of single-target interventions and supports the rationale for multimodal therapeutic strategies aimed at shared upstream mediators.

#### 3.4.1. Apoptosis

Apoptosis is an early and prominent feature of intestinal IRI, particularly affecting enterocytes at the villus tips, where oxygen demand is highest. Ischemia-induced ATP depletion and mitochondrial dysfunction trigger the intrinsic apoptotic pathway through mitochondrial outer membrane permeabilization, cytochrome c release, and activation of caspase-9 and downstream effector caspases such as caspase-3. Reperfusion further amplifies apoptotic signaling via oxidative stress, calcium overload, and activation of pro-apoptotic Bcl-2 family members. Although apoptosis is often considered a non-inflammatory form of cell death, excessive apoptotic loss of epithelial cells compromises barrier integrity and indirectly promotes inflammation by facilitating bacterial translocation [17,18,19,46,47].

#### 3.4.2. Ferroptosis

Ferroptosis has emerged as a critical and previously underappreciated contributor to intestinal epithelial injury during IRI. This iron-dependent form of regulated cell death is driven by uncontrolled lipid peroxidation of polyunsaturated fatty acids within cellular membranes. Key molecular determinants include glutathione peroxidase 4 (GPX4), whose depletion or inactivation permits accumulation of lipid hydroperoxides, and acyl-CoA synthetase long-chain family member 4 (ACSL4), which enhances membrane susceptibility to peroxidation. During reperfusion, excessive reactive oxygen species (ROS) production, mitochondrial dysfunction, and iron accumulation synergistically promote ferroptotic cell death. Importantly, ferroptosis is closely linked to inflammatory signaling, as lipid peroxidation products can activate NF-κB and NLRP3 inflammasome pathways, thereby amplifying local and systemic inflammatory responses [48,49,50,51].

#### 3.4.3. Pyroptosis

Pyroptosis represents an inflammatory form of programmed cell death mediated by inflammasome activation, particularly the NLRP3 inflammasome. In intestinal IRI, danger-associated molecular patterns (DAMPs), mitochondrial ROS, and bacterial products resulting from barrier disruption activate pattern recognition receptors, leading to caspase-1 activation and cleavage of gasdermin D. The formation of gasdermin pores results in cell swelling, membrane rupture, and release of pro-inflammatory cytokines such as interleukin-1β and interleukin-18. Pyroptosis thus serves as a critical link between epithelial injury and innate immune activation, exacerbating mucosal inflammation and systemic cytokine release [52,53,54,55].

#### 3.4.4. Integrated Perspective

Rather than acting as isolated processes, apoptosis, ferroptosis, and pyroptosis operate within a dynamic and interconnected cell death network during intestinal IRI. Oxidative stress and mitochondrial dysfunction function as shared upstream drivers, while inflammatory mediators generated by pyroptosis can further sensitize surrounding cells to apoptotic or ferroptotic death. This convergence of regulated cell death pathways highlights the limitations of single-target therapeutic strategies and supports the rationale for multimodal interventions aimed at upstream triggers such as oxidative stress, mitochondrial injury, and inflammatory amplification [55,56,57,58,59,60].

## 4. Pharmacological Therapeutic Strategies

This section reviews pharmacological interventions targeting oxidative stress, inflammation, mitochondrial dysfunction, and microvascular impairment in intestinal ischemia–reperfusion.

### 4.1. Antioxidants and Redox Modulators

Melatonin, allopurinol, N-acetylcysteine (NAC), and other antioxidants significantly reduce lipid peroxidation, enhance endogenous antioxidant activity, and preserve mitochondrial integrity [24,26,61], leading to improved histopathological outcomes [43].

**Figure 3:**
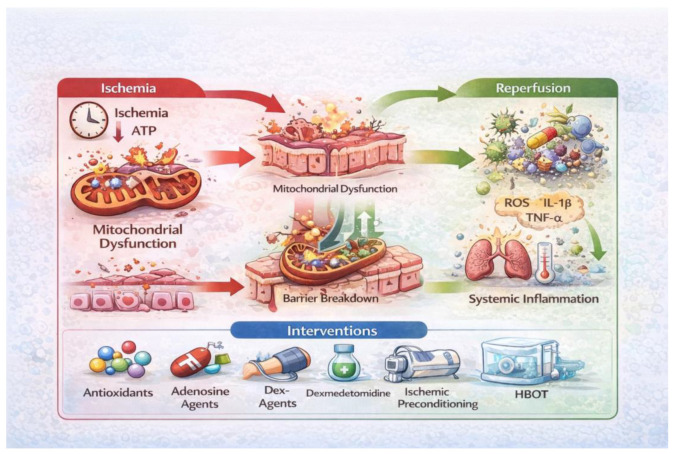
Therapeutic decision map for intestinal ischemia–reperfusion injury. (Conceptual framework integrating temporal injury phases with dominant molecular mechanisms and corresponding therapeutic strategies. The figure illustrates optimal intervention windows and highlights the limitations of single-target approaches, supporting the rationale for multimodal, phase-specific treatment algorithms, executed with the aid of Adobe Photoshop version 26, released October 2024 (accessed online on 1 December).

Oxidative stress-induced damage is mainly caused in the early stages of reperfusion when there is an acute imbalance between the reactive oxygen species and the body’s natural defenses. Consequently, redox-modulating pharmaceuticals and antioxidants have undergone extensive investigation in animal models. N-acetylcysteine, allopurinol, melatonin, and edaravone protect cells by getting rid of free radicals, keeping glutathione levels stable, and lowering lipid peroxidation (Figure 3) [24,62,63]. Figure 3 summarizes a therapeutic decision map linking dominant pathophysiological mechanisms to optimal intervention windows, illustrating why single-target strategies often fail when applied outside their effective temporal context.

In addition to being an antioxidant, melatonin also stimulates mitophagy, activates Nrf2, and stabilizes mitochondrial membranes [43]. These actions maintain villi integrity, mitigate systemic inflammation, and decrease epithelial apoptosis. Edaravone decreases oxidative damage to mitochondria and makes the histology of ischemia/reperfusion in the intestines better [24,25,64].

Despite promising preclinical findings, antioxidant therapy is hard to use because of the dosage, timing, and intricate redox signaling required for tissue defense and regeneration. When used with other treatments, antioxidants can be useful as adjuvants [24,26,65].

### 4.2. Adenosine-Based and Anti-Inflammatory Interventions

Adenosine signaling and anti-inflammatory therapies modulate key immune and vascular pathways involved in intestinal ischemia–reperfusion injury. Pharmacological agents acting on adenosine receptors improve mucosal perfusion, decrease leukocyte adhesion, and mitigate cytokine cascades [27,66].

Post-ischemia–reperfusion (I/R) adenosine signaling is a powerful anti-inflammatory, vasodilatation and cytoprotective defensive mechanism, important for the immune system and microvascular homeostasis management. ATP breakdown and ectonucleotidase increase extracellular adenosine during ischemia. In intestinal ischemia/reperfusion, adenosine-based therapy reduces epithelium damage, sustains microcirculatory flow, and limits systemic inflammation (Figure 3) [27].

Of the four types of adenosine receptors (A_1_, A_2_A, A_2_B, and A_3_), A_2_A and A_2_B are crucial for tissue protection during reperfusion. Activation of A_2_A receptors on endothelial cells, neutrophils, and macrophages reduces pro-inflammatory cytokines, prevents neutrophil adhesion to blood arteries, and boosts nitric oxide-dependent vasodilation. These steps improve capillary perfusion and reduce “no-reflow” after intestinal reperfusion. At a molecular level, activation of the A_2_A receptor inhibits NF-κB signaling and subsequent production of TNF-α, IL-1β, and IL-6. In addition, adenosine signaling promotes cyclic AMP-dependent pathways that prevent activated neutrophils from releasing oxidative bursts, protecting epithelial and endothelial cells from ROS. Pharmacological A_2_A agonists have consistently reduced mucosal necrosis, preserved villus architecture, and improved barrier function in intestinal ischemia/reperfusion models [27].

### 4.3. Dexmedetomidine

Dexmedetomidine has emerged as a promising pharmacological agent due to its combined anti-inflammatory, mitochondrial-protective, and microcirculatory effects with improved mucosal perfusion in animal models [26,32].

Dexmedetomidine, an α_2_-adrenergic agonist, exerts cytoprotective effects with substantial anti-apoptotic, anti-inflammatory, and antioxidant effects by activation of the PI3K/Akt, MAPK, and NF-κB pathways in multiple ischemia–reperfusion injury models (Figure 3) [42,51,67].

Tissue hypoxia and oxidative stress after IRI make microvascular effects especially important [26]. Dexmedetomidine reduces leukocyte-endothelial contact and activation, which improves intestinal microcirculation. Increased nitric oxide bioavailability and decreased VCAM-1 and ICAM-1 increase capillary vasodilation and reduce “no-reflow” following reperfusion [26,32,68,69].

Dexmedetomidine’s clinical efficacy and safety profile benefit translation. Preclinical results show that early reperfusion or perioperative administration boosts its protective qualities, and that it may synergize with antioxidant therapy or conditioning approaches. Targeted clinical research is needed to determine intestinal ischemia–reperfusion damage dosage, timing, and patient selection [32,70].

### 4.4. Microbiota-Targeted Therapies

Probiotics and SCFA donors have shown the capacity to regulate systemic inflammation, enhance epithelial barrier function, and support mucosal healing (Table 3) [30,33].

The intestinal microbiota plays a pivotal role in modulating susceptibility to ischemia–reperfusion injury and in shaping local and systemic inflammatory responses following reperfusion. Intestinal ischemia rapidly disrupts epithelial integrity and microvascular perfusion, leading to dysbiosis characterized by reduced microbial diversity, loss of beneficial commensals, and overgrowth of pathobionts. Upon reperfusion, barrier breakdown facilitates bacterial translocation and release of microbial products such as lipopolysaccharide, which amplify inflammatory signaling through Toll-like receptor and inflammasome pathways. These observations have prompted growing interest in microbiota-targeted therapeutic strategies as adjunctive approaches in intestinal IRI [29,71].

#### 4.4.1. Probiotics and Prebiotics

Probiotics, including Lactobacillus and Bifidobacterium species, have demonstrated protective effects in experimental models of intestinal IRI by enhancing tight junction integrity, reducing oxidative stress, and attenuating pro-inflammatory cytokine production. Prebiotics and dietary fibers further support these effects by promoting short-chain fatty acid (SCFA) production, particularly butyrate, which serves as an energy substrate for enterocytes and exerts anti-inflammatory and barrier-stabilizing properties. Despite encouraging preclinical data, clinical evidence remains limited and heterogeneous, with variability in probiotic strains, dosing, timing, and patient populations contributing to inconsistent outcomes [30,72].

#### 4.4.2. Microbiota Modulation and Immune Crosstalk

Beyond direct barrier protection, microbiota-targeted interventions may influence intestinal IRI through modulation of immune–microvascular interactions. Microbial metabolites regulate endothelial function, leukocyte recruitment, and macrophage polarization, thereby indirectly affecting microcirculatory perfusion and inflammatory amplification. Conversely, microvascular no-reflow and endothelial injury exacerbate dysbiosis by creating hypoxic and nutrient-deprived luminal environments. This bidirectional relationship highlights the need to consider microbiota modulation within a broader framework that integrates vascular protection and immune regulation [31,32].

#### 4.4.3. Fecal Microbiota Transplantation and Emerging Approaches

Fecal microbiota transplantation (FMT) has shown promise in restoring microbial diversity and reducing inflammation in selected gastrointestinal disorders, but its role in intestinal IRI remains largely unexplored. Safety concerns, logistical challenges, and lack of controlled clinical data currently limit its applicability in acute ischemic settings. Emerging strategies, including postbiotics, microbial-derived metabolites, and precision microbiota editing, may offer more controlled and scalable alternatives in the future [33,34].

#### 4.4.4. Translational Limitations and Future Directions

A major limitation of microbiota-targeted therapies in intestinal IRI is the disconnect between experimental models and clinical reality. Animal studies often involve young, otherwise healthy subjects with defined microbial communities, whereas human patients typically present with advanced age, comorbidities, antibiotic exposure, and pre-existing dysbiosis. Moreover, the timing of intervention is critical: microbiota modulation may be more effective in the late reperfusion or recovery phase rather than during acute ischemia. Future research should prioritize stratified approaches that account for baseline microbial composition, integrate microbiota modulation with vascular and anti-inflammatory therapies, and evaluate clinically relevant endpoints [35].

Collectively, microbiota-targeted therapies represent a promising but still underdeveloped component of multimodal strategies for intestinal ischemia–reperfusion injury. Their successful translation will depend on improved mechanistic understanding of microbiota–microcirculation–immune crosstalk and careful alignment of experimental design with clinical contexts [36,37,38].

## 5. Non-Pharmacological and Conditioning Approaches

Non-pharmacological conditioning strategies aim to enhance endogenous protective mechanisms and reduce ischemia–reperfusion-induced intestinal injury

### 5.1. Ischemic Preconditioning (IPC) and Postconditioning (IPoC)

Non-pharmacological methods, including ischemic preconditioning (IPC) and postconditioning (IPoC), exhibit substantial protective effects by activating endogenous defense mechanisms, such as increased nitric oxide production, the induction of heat shock proteins, and reduced production of mitochondrial reactive oxygen species during reperfusion [44], leading to microvascular stabilization, with reduced ROS production and better preservation of the epithelial structure (Figure 3) [24,25].

Ischemic preconditioning involves brief, controlled episodes of ischemia followed by reperfusion before a long episode. In intestinal I/R models, IPC increases post-ischemic microvascular perfusion, decreases epithelial apoptosis, and significantly reduces mucosal damage [30,31,52,54]. IPC modulates mitochondrial permeability transition pore opening, increases nitric oxide (NO) bioavailability via endothelial nitric oxide synthase (eNOS), and upregulates heat shock proteins (HSP70 and HSP90) [54,57].

IPC decreases ROS generation during reperfusion and enhances adaptive redox signaling by activating antioxidant pathways including AMPK and Nrf2 [55,57]. IPC also lowers leukocyte–endothelial interactions and enhances microcirculatory flow by decreasing VCAM-1 and ICAM-1 expression.

### 5.2. Hyperbaric Oxygen Therapy (HBOT)

HBOT consistently improves mucosal oxygenation, reduces neutrophil adhesion, promotes epithelial healing, and attenuates oxidative injury [25,26], thereby acting as a valuable supplemental treatment [45].

Hyperbaric oxygen treatment (HBOT) may enhance tissue oxygenation, reduce inflammation during intestinal ischemia/reperfusion (I/R) and maintain villus architecture after reperfusion in experimental animals [28]. It diminishes leukocyte adhesion and endothelial activation, elevates plasma dissolved oxygen levels, and augments mitochondrial oxidative phosphorylation [17,28]. It also exhibits antioxidant activity by reinstating antioxidant enzyme activity with consequently reduced lipid peroxidation [28].

### 5.3. Controlled Hypothermia and Ex Vivo Perfusion Models

Controlled hypothermia and ex vivo perfusion models provide valuable mechanistic and translational insights into intestinal ischemia–reperfusion injury. Hypothermia limits apoptosis and metabolic demand, while isolated perfused intestinal models reveal detailed mechanistic interactions between microvascular flow, epithelial response, and reperfusion-induced oxidative shifts (Table 4) [22,34].

The ex vivo perfusion model is useful for understanding the molecular mechanisms of early epithelial damage, mitochondrial dysfunction, and barrier failure after reperfusion. Studies employing normo- and hypothermic perfusion techniques show that controlled reperfusion reduces abrupt oxidative bursts, preserves mitochondrial respiratory capacity, and decreases endothelial activation. By evaluating microcirculatory flow, vascular resistance, and oxygen extraction in real time, ex vivo models link perfusion dynamics to tissue survival [22,73].

Ex vivo perfusion systems provide molecular study of signaling pathways involved in oxidative stress, inflammasome activation, and regulated cell death such as necroptosis, pyroptosis, and apoptosis. These systems offer tailored therapies that mechanistically validate therapeutic targets established through in vivo research, such as antioxidant, nitric oxide donor, or metabolic substrate supplementation [34,74].

**Figure 4:**
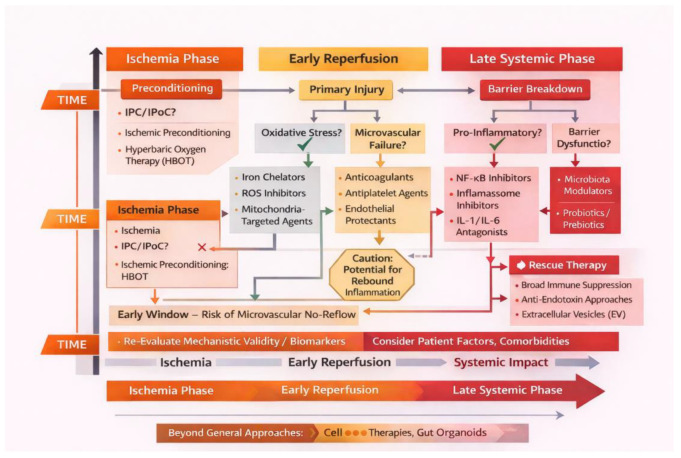
Therapeutic decision map for intestinal ischaemia–reperfusion injury.

Current data supports the fact that single-target therapy is insufficient to tackle the intricate and multidimensional character of IRI. Comprehensive treatment frameworks that include antioxidants, anti-inflammatory drugs, endothelium stabilizers, conditioning methods, and microbiota manipulation are essential to significantly reduce morbidity and mortality linked to intestinal ischemia–reperfusion (Figure 4) [75].

Despite substantial advances in in vivo and ex vivo experimental models that have deepened our understanding of the pathophysiological mechanisms underlying intestinal ischemia–reperfusion injury, translation into effective clinical therapies remains limited. A major barrier to translation arises from the pronounced heterogeneity of experimental models, including wide variability in ischemia duration, reperfusion intervals, temperature control, animal species and age, anesthetic protocols, and outcome measures [76]. These methodological discrepancies not only impair reproducibility across studies but also complicate the extrapolation of experimental findings to clinically relevant scenarios, where ischemic insults are highly variable and frequently accompanied by comorbidities and systemic inflammation [46,77,78].

In addition, many experimental studies rely on short-term surrogate endpoints—such as histological injury scores, oxidative stress markers, or cytokine expression—without assessing longer-term functional recovery, barrier restoration, or systemic consequences. This focus may overestimate therapeutic efficacy in controlled experimental settings while failing to capture clinically meaningful outcomes. Collectively, these limitations underscore the urgent need for greater standardization of experimental protocols, including harmonized definitions of ischemia severity, reperfusion timing, and core outcome measures, as well as broader adoption of ex vivo perfusion platforms that more closely mimic human physiology [53,54].

Another critical translational challenge lies in the predominant evaluation of single-target therapies, despite the inherently multifactorial nature of intestinal IRI. Oxidative stress, endothelial dysfunction, inflammatory amplification, epithelial barrier breakdown, and microbiota dysregulation do not occur in isolation but evolve dynamically and interact across distinct temporal phases of injury. Therapeutic strategies that selectively modulate only one pathway may therefore provide partial or transient protection, which helps explain the limited success of many interventions when advanced toward clinical testing [79,80].

These observations collectively support a paradigm shift toward multimodal and phase-specific therapeutic approaches, integrating interventions that simultaneously restore redox homeostasis, preserve endothelial and microvascular integrity, modulate innate immune responses, and stabilize the intestinal microbiota. Importantly, such strategies must also account for therapeutic timing, as interventions effective during ischemia or early reperfusion may differ from those required to mitigate late systemic inflammation and organ dysfunction. Addressing these translational barriers will be essential for converting mechanistic insights into clinically impactful therapies for intestinal ischemia–reperfusion injury [81].

A recurring theme across the literature is the discrepancy between experimental efficacy and clinical performance of therapeutic interventions for intestinal ischemia–reperfusion injury. While numerous agents are described as “promising” based on animal studies, relatively few have progressed to clinical evaluation, and even fewer have demonstrated reproducible benefit. This imbalance reflects not only biological complexity but also methodological limitations, including model selection bias and overinterpretation of short-term outcomes. Recognizing these limitations is essential to avoid perpetuating a cycle of translational optimism unsupported by clinical evidence [79].

Importantly, ex vivo perfusion models may serve as a valuable intermediate platform by allowing controlled manipulation of perfusion parameters while preserving tissue architecture. However, even these systems lack the systemic immune and neurohumoral influences present in human disease, reinforcing the necessity of cautious interpretation [80].

Future progress will depend on comprehensive, multimodal therapy strategies that include antioxidant support, endothelial protection, immunological modulation, microbiome restoration, and ischemia conditioning.

### 5.4. Translational Barriers and Evidence Hierarchy in Intestinal Ischemia–Reperfusion Injury

Despite extensive preclinical investigation, effective clinical translation of therapeutic strategies for intestinal ischemia–reperfusion injury remains limited. A major contributor to this gap is the heterogeneity of experimental models, encompassing differences in ischemia duration, reperfusion timing, temperature control, animal species, and outcome measures. Such variability complicates reproducibility and limits direct extrapolation to clinical scenarios, where ischemic insults are often unpredictable, prolonged, and accompanied by comorbidities and systemic inflammation. Importantly, evidence derived from in vitro and small-animal models is frequently interpreted alongside limited clinical data without sufficient differentiation of evidentiary strength [75,76].

In addition, many experimental interventions are administered either prior to ischemia or at the onset of reperfusion, conditions that rarely reflect clinical reality. This timing mismatch may partially explain why therapies demonstrating robust protective effects in controlled experimental settings fail to show benefit in patients who typically present after injury has already progressed. Furthermore, the frequent reliance on short-term surrogate endpoints—such as histological injury scores, oxidative stress markers, or cytokine expression—may overestimate therapeutic efficacy while neglecting longer-term outcomes such as functional recovery, barrier restoration, and systemic complications [77].

Collectively, these factors underscore the need for a hierarchical interpretation of evidence that clearly distinguishes between preclinical, ex vivo, and clinical findings, and for translational pipelines that incorporate clinically relevant timing, endpoints, and patient heterogeneity.

Conceptual figures presented in this review are intended to reframe intestinal ischemia–reperfusion injury as a temporally evolving and mechanistically interconnected process rather than a static collection of pathways. By integrating existing evidence into temporal and decision-oriented models, these figures help explain why therapies targeting isolated mechanisms frequently fail when applied without consideration of injury phase or mechanistic dominance. This conceptual reorganization provides a framework for future experimental design and translational research without relying on additional experimental data.

### 5.5. Structural Reasons for Translational Failure in Intestinal Ischemia–Reperfusion Injury

The repeated failure of experimental advances to translate into effective clinical therapies for intestinal ischemia–reperfusion injury reflects a fundamental mismatch between experimental controllability and clinical uncertainty. In laboratory models, ischemia onset, duration, and reperfusion are precisely defined, body and ambient temperature are tightly regulated, and confounding variables are intentionally minimized—conditions that create a level of temporal and physiological certainty unattainable in human in vivo conditions. In contrast, patients with acute mesenteric ischemia rarely present with a clearly defined onset of ischemia, and no validated biomarker or imaging modality currently exists to accurately determine ischemic duration, severity, or reversibility at the bedside [82,83]. This temporal ambiguity critically undermines phase-specific experimental interventions, which are often applied at biologically optimal but clinically unrealistic time points [84]. Furthermore, human intestinal ischemia occurs in the context of advanced age, comorbid vascular disease, systemic inflammation, and heterogeneous microbiota, all of which profoundly modify injury trajectories and therapeutic responsiveness [83,84]. As a result, experimental therapies that appear robust in controlled models frequently fail when confronted with the biological noise and unpredictability of real-world clinical settings. Although this translational disconnect is well recognized by clinicians, the field still lacks a feasible experimental or clinical study design capable of capturing ischemic uncertainty and reversibility in humans, particularly in the setting of critical yet potentially salvageable intestinal ischemia.

A realistic path toward improving translation would require experimental designs that intentionally incorporate uncertainty rather than eliminating it. Instead of relying on a single, fixed ischemia duration, future models should employ variable and nonuniform ischemic windows, with the exact onset of ischemia blinded to the investigators administering therapy, thereby forcing intervention decisions to be based on measurable physiological or biochemical triggers rather than predefined time points. Such triggers could include hemodynamic instability, metabolic markers, indices of microcirculatory failure, or early indicators of barrier dysfunction. In parallel, models should incorporate clinically relevant comorbidities, such as advanced age, vascular disease, metabolic dysfunction, or low-grade systemic inflammation, and should prioritize endpoints with direct clinical relevance, including barrier recovery, bacterial translocation, remote organ dysfunction, and medium-term survival. A staged translational pipeline—combining mechanistic screening in small-animal models, validation in ex vivo perfusion systems with controlled reperfusion parameters and delayed-intervention testing in large-animal models—may provide a more faithful approximation of human intestinal ischemia–reperfusion injury. Ultimately, such an approach would allow the field to test the central but largely unproven clinical assumption that translational failure arises not from the absence of actionable molecular targets, but from the profound temporal and biological uncertainty inherent to human ischemic disease.

## 6. Future Directions

Novel omics-based methods (proteomics, transcriptomics, metabolomics) provide essential tools for clarifying the molecular markers of IRI and for identifying novel treatment targets. Proteomic profiling has shown significant alterations in cytoskeletal components, mitochondrial enzymes, and heat shock proteins, suggesting possible novel targets for therapeutic interventions [47]. The real-time evaluation of microcirculation using techniques like fluorescence angiography or side stream dark-field imaging is likely to improve perioperative decision-making and facilitate the customization of interventions according to individual pathophysiological profiles [54].

Also, the employment of specific phase biomarkers can improve therapeutic interventions, since in the first postischemic phase, there is high ROS production, which could benefit from preconditioning, HBOT antioxidants, and inhibitors of apoptosis/ferroptosis, while in the second reperfusion phase, there is a more pronounced adenosine signaling and inflammation, which could be treated more efficiently by inhibitors of adenosine and anti-inflammatory drugs (Figure 4). However, the temporal characterization of pathological events is particularly challenging in the complex clinical setting of ischemia–reperfusion injury. Consequently, there is a strong need to identify reliable biomarkers that enable accurate staging and longitudinal monitoring of disease progression, as well as the elucidation of the underlying pathophysiological mechanisms involved.

Future research in intestinal ischemia–reperfusion injury should prioritize the development of more accurate and standardized experimental models that recapitulate human physiology. Robust evaluation of IRI management strategies requires harmonized testing protocols that integrate ex vivo systems, in vivo models, and human preclinical studies, to eventually achieve effective clinical translation.

Current ex vivo and in vivo models often fail to capture the complexity of human intestinal IRI, particularly concerning gut microbiota dysbiosis, dysregulated immune responses, and the progression from localized intestinal inflammation to systemic inflammatory response syndrome (SIRS). Moreover, species-specific physiological differences limit the translatability of animal data to human outcomes. Considering these limitations and growing ethical concerns regarding animal experiments, there is an urgent need to implement alternative and complementary testing strategies, such as advanced organ-on-chip models, human-containing tissue models, and computational approaches, that reduce reliance on animal models while maintaining scientific rigor and compliance with regulatory requirements.

Future research should prioritize strategies that explicitly address translational barriers rather than solely expanding the repertoire of experimental targets. This includes the development of standardized experimental protocols, stratification of therapeutic efficacy according to ischemia severity and timing, and adoption of clinically relevant endpoints. Multimodal therapeutic approaches should be evaluated in staged designs that reflect the temporal evolution of injury, distinguishing early ischemic, reperfusion, and late systemic phases. Moreover, negative and neutral findings should be reported with equal rigor to prevent overestimation of therapeutic potential.

Ultimately, advancing the field will require a shift from isolated mechanistic validation toward integrated translational frameworks that align experimental design with clinical reality.

## 7. Conclusions

Intestinal ischemia–reperfusion injury remains a major clinical challenge in abdominal surgery, intestinal transplantation, and acute mesenteric ischemia, with limited effective therapeutic options despite extensive experimental investigation. The persistent gap between mechanistic understanding and clinical benefit underscores the need to reconsider how experimental insights are translated into therapeutic strategies.

From a clinical perspective, this review highlights that intestinal IRI should not be approached as a uniform pathological entity, but rather as a temporally evolving process in which dominant mechanisms differ across ischemic, early reperfusion, and late systemic phases. Recognizing this temporal heterogeneity has important implications for clinical management, as interventions effective at one stage may be ineffective or even detrimental at another. Therapeutic timing, patient stratification, and injury severity must therefore be integrated into clinical decision-making.

Future therapeutic development should prioritize multimodal, phase-specific strategies that simultaneously address interconnected injury domains, including oxidative stress, microvascular dysfunction, epithelial barrier failure, inflammatory amplification, and microbiota dysregulation. Single-target interventions are unlikely to provide durable benefit in isolation, whereas coordinated approaches tailored to specific injury phases may offer greater translational potential.

Advances in precision biomarkers represent a critical opportunity to improve patient stratification and therapeutic targeting. Biomarkers reflecting redox status, endothelial injury, mitochondrial dysfunction, regulated cell death pathways, and microbial translocation may enable real-time assessment of injury phase and guide personalized intervention strategies. Integration of such biomarkers into clinical protocols could enhance both therapeutic efficacy and patient safety.

Finally, future translational research should move beyond expanding the list of experimental targets and instead focus on standardized, clinically relevant models, harmonized outcome measures, and rigorous evaluation of negative as well as positive findings. Greater emphasis on ex vivo platforms, multimodal intervention designs, and alignment of experimental timing with clinical reality will be essential to overcome current translational barriers. By adopting these principles, the field may progress toward more effective and clinically meaningful therapies for intestinal ischemia–reperfusion injury.

## Figures and Tables

**Table 1:** Oxidative and inflammatory pathways activated during IRI.

Mechanistic Domain	Key Findings	Molecular/Cellular Markers
Microvascular dysfunction	Capillary no-reflow; endothelial swelling; perfusion failure	ICAM-1 ↑, VCAM-1 ↑, P-selectin ↑, eNOS ↓
Endothelial glycocalyx degradation	Increased permeability; leukocyte adhesion	Syndecan-1 ↑, Heparan sulfate fragments ↑
Mitochondrial injury	Cristae disruption; ROS burst; impaired OXPHOS	MDA ↑, SOD ↓
Tight junction disruption	Loss of barrier integrity; bacterial translocation	Occludin ↓, Claudin-1 ↓, ZO-1 ↓
Apoptosis/Pyroptosis	Enterocyte apoptosis and pyroptosis	Caspase-3 ↑, Bax ↑, Bcl-2 ↓, NLRP3 ↑

Note: ↓—decreased levels; ↑—increased levels.

**Table 2:** Molecular oxidative and inflammatory pathways activated in intestinal ischemia–reperfusion injury.

Pathway	Molecular Events	Consequences	Key References
NF-κB Activation	ROS → IκB degradation → NF-κB nuclear translocation	TNF-α ↑, IL-1β ↑, IL-6 ↑	[25,26]
Nrf2 Suppression	Reduced antioxidant response	SOD ↓, CAT ↓, GSH/GSSG imbalance	[27]
MAPK Pathway	ROS and cytokine activation	Apoptosis, inflammation	[28,29]
TLR4–NLRP3 Inflammasome	TLR4 activation → caspase-1 activation	Pyroptosis, IL-18 ↑	[30,31]
Nitrosative Stress	NO–peroxynitrite formation	Endothelial injury, lipid peroxidation	[32,33]

Note: ↓—decreased levels; ↑—increased levels.

**Table 3:** Therapeutic interventions in intestinal IRI.

Therapeutic Class	Representative Agents/Strategies	Primary Molecular Targets/Mechanisms	Key Experimental or Clinical Effects	Stage of Clinical Development
Antioxidants	N-acetylcysteine (NAC), melatonin, edaravone	ROS scavenging; Nrf2 activation; mitochondrial protection	↓ Lipid peroxidation; ↓ apoptosis; preserved villus architecture	Preclinical; several agents clinically approved for other indications
Adenosine-based therapies	Adenosine A_2_A/A_2_B receptor agonists	NF-κB inhibition; vasodilation; anti-inflammatory signaling	Improved microcirculation; ↓ cytokine release	Preclinical; early clinical studies
α_2_-Adrenergic agonists	Dexmedetomidine	PI3K/Akt activation; mitochondrial stabilization	Barrier protection; ↓ inflammation	Clinically approved (sedation); preclinical I/R evidence
Anti-inflammatory agents	Cytokine inhibitors; NLRP3 inhibitors	Suppression of inflammatory signaling	↓ TNF-α, IL-1β; ↓ tissue injury	Preclinical
Ferroptosis-targeted therapies	Iron chelators; GPX4 activators	Inhibition of lipid peroxidation	Preserved epithelial integrity	Preclinical/proof-of-concept
Microcirculatory modulators	Nitric oxide donors	Endothelial protection	Improved oxygen delivery	Preclinical
Barrier-protective strategies	Probiotics; SCFAs	Tight junction stabilization	↓ Permeability; ↓bacterial translocation	Preclinical; limited early clinical data

Note: ↓—decreased levels.

**Table 4:** Non-pharmacological strategies in IRI.

Strategy	Mechanistic Basis	Protective Effects	References
Ischemic Preconditioning (IPC)	Brief ischemia → HSPs ↑, NO ↑	↓ Mucosal injury, improved microcirculation	[19,68]
Ischemic Postconditioning (IPoC)	Intermittent reperfusion cycles	↓ ROS burst, ↓ apoptosis	[23,41]
Hyperbaric Oxygen Therapy	↑ Oxygen pressure; ↓ neutrophil adhesion	Improved villus structure; ↓ Oxidative stress	[6,7,69]
Hypothermia	Reduced metabolic demand	Preserves mucosa and mitochondria	[57,70]
Ex Vivo Perfusion Models	Controlled ischemia/reperfusion	Mechanistic insights without systemic variables	[10,72]

Note: ↓—decreased levels; ↑—increased levels.

## Data Availability

All data supporting the findings of this review are derived from previously published articles, which are cited within the manuscript. All figures are original work; copyright can be obtained on demand from the corresponding author. No new data were created or analyzed in this study.

## Abbreviations

Akt	Protein kinase B
AP-1	Activator protein 1
ATP	Adenosine triphosphate
A2AR	Adenosine A_2_A receptor
BBB	Blood–brain barrier
Bcl-2	B-cell lymphoma 2
Bax	Bcl-2-associated X protein
CAT	Catalase
cGAS	Cyclic GMP–AMP synthase
Chiu Score	Histological injury grading system for intestinal I/R
CLDN-1	Claudin-1
COX	Cyclooxygenase
CXCL	C-X-C motif chemokine ligand
DAMPs	Damage-associated molecular patterns
DEX	Dexmedetomidine
DNA	Deoxyribonucleic acid
ECM	Extracellular matrix
eNOS	Endothelial nitric oxide synthase
ER	Endoplasmic reticulum
FABP2	Fatty acid binding protein 2 (enterocyte injury marker)
FRAP	Ferric reducing antioxidant power
GSH	Reduced glutathione
GSH-Px	Glutathione peroxidase
GSH/GSSG	Reduced/oxidized glutathione ratio
GI	Gastrointestinal
HBOT	Hyperbaric oxygen therapy
H&E	Hematoxylin and eosin staining
HIF-1α	Hypoxia-inducible factor 1-alpha
HSP70/HSP90	Heat shock proteins 70/90
ICAM-1	Intercellular adhesion molecule-1
IL-1β, IL-6, IL-18	Interleukins 1β, 6, and 18
IPC	Ischemic preconditioning
IPoC	Ischemic postconditioning
IRI	Ischemia–reperfusion injury
I/R	Ischemia–reperfusion
iNOS	Inducible nitric oxide synthase
LDH	Lactate dehydrogenase
LPS	Lipopolysaccharide
LTA	Lipoteichoic acid
MDA	Malondialdehyde
MAPK	Mitogen-activated protein kinase
MLCK	Myosin light-chain kinase
MODS	Multiple organ dysfunction syndrome
MPO	Myeloperoxidase
NADPH oxidase	Nicotinamide adenine dinucleotide phosphate oxidase
NF-κB	Nuclear factor-κB
NLRP3	NOD-like receptor family pyrin domain containing 3
NO	Nitric oxide
NOS	Nitric oxide synthase
Nrf2	Nuclear factor erythroid 2-related factor 2
Occludin (OCLN)	Tight junction protein
OXPHOS	Oxidative phosphorylation
PI3K	Phosphoinositide 3-kinase
PRISMA	Preferred Reporting Items for Systematic Reviews and Meta-Analyses
PAMPs	Pathogen-associated molecular patterns
PGE_2_	Prostaglandin E_2_
RNS	Reactive nitrogen species
ROS	Reactive oxygen species
RNA	Ribonucleic acid
SCFAs	Short-chain fatty acids
SIRS	Systemic inflammatory response syndrome
SMA	Superior mesenteric artery
SOD	Superoxide dismutase
TJ	Tight junctions
TLR	Toll-like receptor
TNF-α	Tumor necrosis factor-alpha
UC	Ulcerative colitis (contextual comparison)
VCAM-1	Vascular cell adhesion molecule-1
VEGF	Vascular endothelial growth factor
